# IMPACTS OF COVID-19 ON TRAINING HEALTH CARE WORKERS AT EAST LOS ANGELES COMMUNITY COLLEGE

**DOI:** 10.1093/geroni/igad104.1692

**Published:** 2023-12-21

**Authors:** Janice Velazquez

**Affiliations:** East Los Angeles College, Monterey Park, California, United States

## Abstract

The Pandemic changed how community colleges prepare the next generation of healthcare workers, and they are slowly adjusting to remain relevant in the current environment. How community colleges train their students will determine how well their communities will be cared for, which is why this topic is important. Some of the strategies implemented to fulfill the East Los Angeles’ College (ELAC) workforce demands following COVID are: aligning basics skills with some of the courses; providing career counseling, including mock interviews; opportunities to branch out and transfer in two years; connecting students with services to address their basic needs including mental health. Faculty are also required to stay current, and they need support from their local Districts and national collaborators. Some completed specialized training in humanizing curriculum and transforming faculty learning programs to improve undergraduate STEM faculty’s instructional practice. Moreover, the ELAC faculty felt that developing a humanities course tailored to Health and Human Services could be a great start to connect current needs with our academic programming. Faculty are revising and collecting content to address recent changes impacting our society and how we operate in the fast-changing work environment. After the Pandemic, the use of technology is more common and accepted. This change facilitates interactions, and this is a contributing factor to the program development process. For example, the advisory questionnaire is now available electronically, and therefore expecting more participation. Faculty have more variety and options for training to improve content development and delivery strategies.

